# *In Silico *Quantitative Structure-Activity Relationship Studies on P-gp Modulators of Tetrahydroisoquinoline-Ethyl-Phenylamine Series

**DOI:** 10.1186/1472-6807-11-5

**Published:** 2011-01-26

**Authors:** Changdev G Gadhe, Thirumurthy Madhavan, Gugan Kothandan, Seung Joo Cho

**Affiliations:** 1 Departments of Bio-New Drug Development, College of Medicine, Chosun University, 375 Seosuk-dong, Dong-gu, Gwangju 501-759, Korea; 2 Cellular-Molecular Medicine and Research Center for Resistant Cells, College of Medicine, Chosun University, 375 Seosuk-dong, Dong-gu, Gwangju 501-759, Korea

## Abstract

**Background:**

Multidrug resistance (MDR) is a major obstacle in cancer chemotherapy. The drug efflux by a transport protein is the main reason for MDR. In humans, MDR mainly occurs when the ATP-binding cassette (ABC) family of proteins is overexpressed simultaneously. P-glycoprotein (P-gp) is most commonly associated with human MDR; it utilizes energy from adenosine triphosphate (ATP) to transport a number of substrates out of cells against concentration gradients. By the active transport of substrates against concentration gradients, intracellular concentrations of substrates are decreased. This leads to the cause of failure in cancer chemotherapy.

**Results:**

Herein, we report Topomer CoMFA (Comparative Molecular Field Analysis) and HQSAR (Hologram Quantitative Structure Activity Relationship) models for third generation MDR modulators. The Topomer CoMFA model showed good correlation between the actual and predicted values for training set molecules. The developed model showed cross validated correlation coefficient (*q*^2^) = 0.536 and non-cross validated correlation coefficient (*r*^2^) = 0.975 with eight components. The best HQSAR model (*q*^2 ^= 0.777, *r*^2 ^= 0.956) with 5-8 atom counts was used to predict the activity of test set compounds. Both models were validated using test set compounds, and gave a good predictive values of 0.604 and 0.730.

**Conclusions:**

The contour map near R1 indicates that substitution of a bulkier and polar group to the ortho position of the benzene ring enhances the inhibitory effect. This explains why compounds with a nitro group have good inhibitory potency. Molecular fragment analyses shed light on some essential structural and topological features of third generation MDR modulators. Fragments analysis showed that the presence of tertiary nitrogen, a central phenyl ring and an aromatic dimethoxy group contributed to the inhibitory effect. Based on contour map information and fragment information, five new molecules with variable R1 substituents were designed. The activity of these designed molecules was predicted by the Topomer CoMFA and HQSAR models. The novel compounds showed higher potency than existing compounds.

## Background

MDR, the principal mechanism by which many cancers develop resistance to chemotherapy drugs, is a major factor in the failure of many forms of chemotherapy [1]. In MDR tumour cells, various member of the ABC family of transport proteins can simultaneously be overexpressed: these include P-gp (ABCB1), breast cancer resistance protein (BCRP, ABCG2) and MDR associated protein 1 (MRP1, ABCC family) [2]. These transporters utilize energy from ATP hydrolysis to transport a wide variety of substances out of cells against concentration gradients. The active efflux of substances from cells decreases their intracellular concentration and results in failure of chemotherapy.

Among the 49 identified human ABC transporters, P-gp is most intensively studied [3], and is a member of MDR/TAP (transporter associated proteins) subfamily. P-gp has ability to transport a wide variety of structurally unrelated substances out of cells [4-6]. P-gp is extensively distributed and expressed in the intestinal epithelium, hepatocytes, renal proximal tubular cells, adrenal gland and capillary endothelial cells comprising the blood-brain and blood-testis barrier. P-gp transports structurally diverse substrates and most are anticancer drugs such as doxorubicin, daunorubicin, paclitaxel, etoposid, teniposid, vinblastine and vincristine [7]. P-gp does not interact with anionic compounds but does interact with amphipathic compounds with molecular masses between 400-1900 daltons [8,9]. The calcium channel blocker verapamil can overcome MDR in cancer cells [10]. Another drug, cyclosporine-A, was designed as an immunosuppressant, but shows a promising P-gp inhibitory effect. Both these drugs are used as first generation P-gp inhibitors, also called MDR modulators. The use of these modulators has been limited because of low efficacy and higher dose-related toxicity. The second generation modulators, dexverapamil and PSC833, had higher efficacy and lower toxicity, but produce serious drug-drug interactions clinically.

Nowadays, a third-generation of MDR modulators are under investigation. These drugs, which include tariquidar (XR9576), zosuquidar (LY335979) and laniquidar (R1010933), possess selectivity, low toxicity and high efficacy [11]. These modulators are structurally different from the first- and second-generation modulators. Early in their evaluation, these modulators displayed promising activity. However, toxicity was subsequently observed [12]. The toxicities were found not to be mechanism based. These modulators showed some potential as new drugs, but were dropped due to toxicity related to high dose to be effective physiologically. Therefore, there is a clear need to enhance the activity of modulators that would also reduce the required dose. The search for new nontoxic, efficacious, potent modulators without drug-drug interactions has been intensive. The studies include a 3D-QSAR and free Wilson analysis on a series of tariquidar analogues as MDR modulators [13], a QSAR study on anthranilamide derivatives containing the nucleus of XR9576 and a 3D-QSAR study using tariquidar derivatives (WK-X and WK-Y compounds) to develop QSAR CoMFA/CoMSIA models [14]. With the aim to find out important groups and atoms for P-gp antagonism, the present study selected a third-generation MDR modulator to develop Topomer CoMFA and HQSAR models [15]. This series consisted of same tetrahydroisoquinoline-ethyl-phenyl-amine nucleus present in two of the aforementioned series.

## Methods

### Data Set

The activity dataset, which was selected from reported literature [15], consisted of 39 molecules (Table 1). For analysis, the given inhibitory concentration values were changed to minus logarithmic scale value (pIC_50_), as a dependent variable for Topomer CoMFA and HQSAR analysis by using the formula provided below. It is common to convert the biological activity data into a logarithmic scale, because the resulting model behaves more reasonably. This would usually give better linear models.

**Table 1:** Structures and biological activities of the dataset compounds.

Compound	**R**_**1**_	**R**_**2**_	X	**pIC**_**50**_
4	OCH_3_	Phenyl	-	5.39
5a*	OCH_3_	2-Nitrophenyl	-	5.27
5b	H	2-Nitrophenyl	-	4.85
6a	OCH_3_	2-Aminophenyl	-	5.07
6b*	H	2-Aminophenyl	-	5.00
7a	OCH_3_	4-Nitrophenyl	-	5.85
7b	H	4-Nitrophenyl	-	5.25
8a	OCH_3_	4-Aminophenyl	-	5.32
8b	H	4-Aminophenyl	-	4.92
9	OCH_3_	3-Quinolinyl	-	6.24
9b	H	3-Quinolinyl	-	6.37
10*	OCH_3_	2-Quinolinyl	-	6.07
11	OCH_3_	4-Quinolinyl	-	5.33
12	OCH_3_	6-Quinolinyl	-	6.17
13	OCH_3_	2-Quinoxalinyl	-	6.33
14	OCH_3_	1-Naphthyl	-	5.85
15*	OCH_3_	2-Naphthyl	-	6.20
16	OCH_3_	3-Pyridyl	-	5.32
17	OCH_3_	2-Bromophenyl	-	5.48
18	OCH_3_	3-Bromophenyl	-	5.74
19	OCH_3_	4-Bromophenyl	-	5.62
20	OCH_3_	3,4-Dimethoxyphenyl	-	5.38
21	OCH_3_	4,5-Dimethoxy-2-nitrophenyl	-	4.89
22	OCH_3_	3,4-Methylendioxyphenyl	-	5.68
23*	OCH_3_	Phenyl	-CH=CH-	5.85
24*	OCH_3_	2-Nitrophenyl	-CH=CH-	5.96
25	OCH_3_	4-Chlorophenyl	-CH=CH-	6.17
26	OCH_3_	4,5-Dimethoxy-2-nitrophenyl	-CH=CH-	5.82
27	OCH_3_	Phenyl	-CH_2_-O-	5.51
28	OCH_3_	2-Nitrophenyl	-CH_2_-O-	6.66
29*	OCH_3_	2-Aminophenyl	-CH_2_-O-	5.47
30a	OCH_3_	2-Nitrophenyl	-NH-	6.48
30b	H	2-Nitrophenyl	-NH-	6.11
31	OCH_3_	3-Nitrophenyl	-NH-	6.17
32	OCH_3_	4-Nitrophenyl	-NH-	5.82
33a	OCH_3_	2-Aminophenyl	-NH-	4.68
33b*	H	2-Aminophenyl	-NH-	4.72
34	OCH_3_	3-Aminophenyl	-NH-	4.85
35	OCH_3_	4-Aminophenyl	-NH-	4.52

Asterisk (*) indicates test set compounds.

$${\text{pIC}}_{\text{5}0}=-{\text{log (IC}}_{\text{5}0}\text{)}\;$$

The dataset was randomly partitioned into training and test set molecules by considering range of molecules (pIC_50 _= 4.52-6.66), so that both the training and test sets consist of high, medium and low activity molecules. The training and test set consist of 31 and 8 molecules, respectively. All the molecules were built using the SYBYL 8.1 molecular modeling package [16]. All the dataset molecules were sketched by the SYBYL sketching program and were minimized by using the Tripos force field. They were then subjected to simulated annealing to get a stable conformation. Simulated annealing was performed for each ligand up to 200 cycles with default parameters, and then conformations were sorted according to the least potential energy value. These conformations were minimized with quantum mechanical semi-empirical AM1 method with precise convergence and full optimization commands with MMOK (Molecular Mechanics Correction to CONH Bonds) keywords. The dataset was then used for Topomer CoMFA and HQSAR analysis.

### Topomer CoMFA

**Figure 1:**
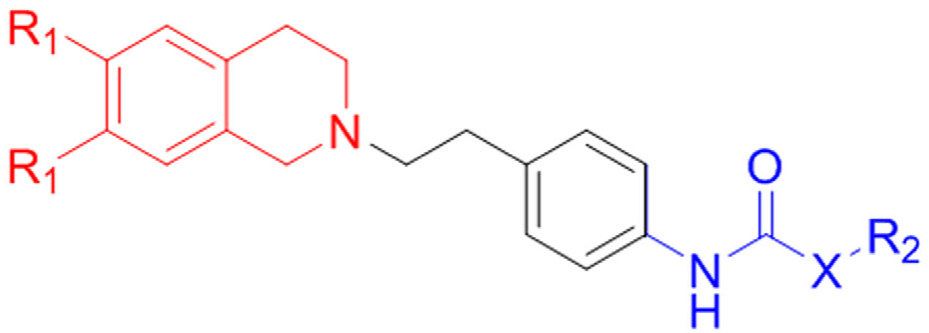
**Fragmentation pattern (R1 and R2) for all molecules of dataset in topomer CoMFA analysis**. R1 fragment is represented by the blue color and R2 fragment is denoted by the red color.

A Topomer CoMFA technique merges CoMFA [17] and topomer technology, to overcome the alignment problem of CoMFA [18]. Topomer CoMFA includes alignment of structural fragments. Structural fragments by definition contain a common feature, the "open valence" or "attachment bond". The Topomer methodology overlaps this common feature to provide an absolute orientation for any fragment. A Topomer is an invariant three-dimensional (3D) representation of molecular subunit generated from its two-dimensional (2D) topology by topomer alignment in topomer CoMFA [19]. In Topomer CoMFA analysis, all molecules of dataset were divided into two fragments, shown as R1 (blue) and R2 (red) groups in Figure 1. Each Topomer fragment was applied with topomer alignment to make a 3D invariant representation [20]. In Topomer CoMFA, atomic charges were calculated by the Gasteiger-Marsilli method for the topomer structure. Topomer CoMFA acts in two different ways for the calculation of molecular fields. An 'attenuation factor' reduces the field contributions of fragment atoms more distant from the attachment bond. Finally, the *r*^2 ^is calculated by using the same optimum number of component obtained from leave-one-out (LOO) cross validation analysis. Topomer CoMFA steric and electrostatic fields were calculated at a regular space grid of 2 Å, and were fixed automatically into a 1000 point cube to contain a Topomer. A sp^3 ^hybridized carbon atom was used as a probe atom for the steric field calculation and a negative oxygen atom was used as a probe for electrostatics field.

### Partial Least Square (PLS)

The relationship between structural parameters and biological activities of compounds under study has been quantified using a PLS algorithm [21-23]. Topomer CoMFA descriptors were used as independent variable and biological activity (pIC_50_) as a dependent variable. The cross-validation analysis was performed by using the LOO method, in which one molecule is removed from the dataset and its activity is predicted by using the model derived from rest of the molecules in the dataset. The *q*^2 ^resulted in an optimum number of components and the lowest standard error of prediction. The *q*^2 ^is calculated using the following equation:

$$q^{2}=1-\frac{{\sum_{\gamma }\left(\gamma_{pred}-\gamma_{actual}\right)}^{2}}{\sum_{\gamma }{\left(\gamma_{actual}-\gamma_{mean}\right)}^{2}}$$

where γ_pred_, γ_actual _and γ_mean _are predicted, actual and mean values of the target property (pIC_50_), respectively.

### Predictive Correlation Coefficient (*r*^2^_*pred*_)

The predictive power of Topomer CoMFA and HQSAR models were derived from set of eight molecules, which were excluded during model development. In the structural preparation of test set molecules, sketching and optimization was same as the training set molecules. The activity of the test set was predicted by using model derived from training set. The predictive correlation coefficient for developed model was determined by using following formula:

$$r_{pred}^{2}=\frac{\left(SD-PRESS\right)}{SD}$$

where, PRESS is the sum of the squared deviation between the predicted and actual activity of the test set molecules, and SD is defined as the sum of the square deviation between the biological activity of the test set compounds and the mean activity of the training set molecules.

### HQSAR

HQSAR is a technique that employs fragment fingerprints as predictive variables of biological activity or other structural related data [24]. HQSAR does not require a 3D structure of bioactive conformation or molecular alignments. HQSAR model generation deals with the 2D structure directed fragment fingerprints [25]. These molecular fingerprints are broken into strings at fixed intervals as specified by a hologram length (HL) parameter. The HL determines the number of bins in the hologram into which the fragments are hashed. The optimal HQSAR model was derived from screening through the 12 default HL values, which were a set of 12 prime numbers ranging from 53-401. The model development was performed using the following parameters: atom (A), bond (B), connection (C), chirality (Ch), hydrogen (H) and donor/acceptor (DA). The validity of the model depends on statistical parameters such as *r*^2^, *q*^2 ^by LOO, predictive *r*^2^_*pred *_and standard error. The robustness of the model depends on the more challenging test set prediction reflected by its predictive *r*^2^_*pred *_value.

## Results and Discussion

### Topomer CoMFA Model Analysis

**Figure 2:**
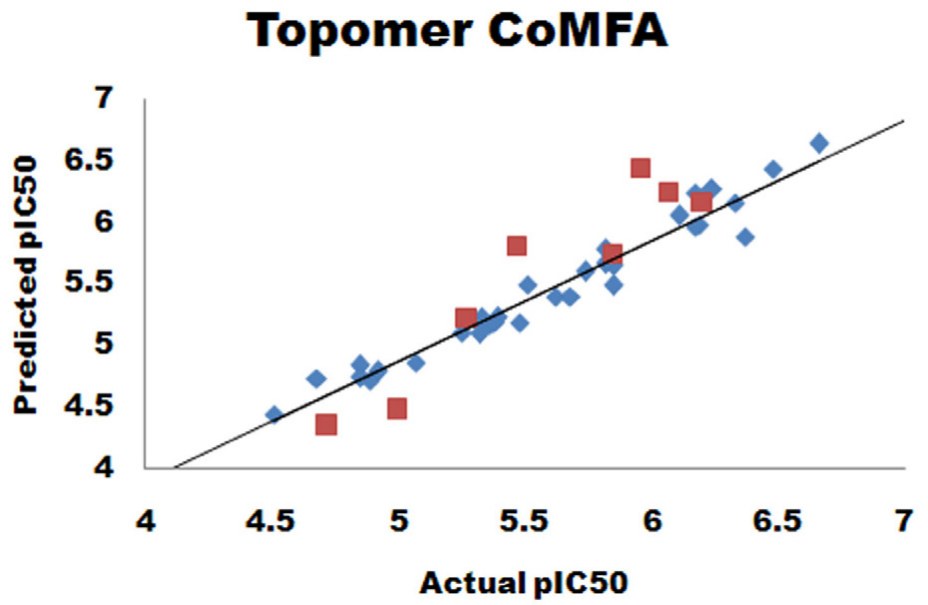
**Scatter plot diagram for Topomer CoMFA analysis**. Predicted versus actual activity of the training set (blue diamond) and the test set (red square) compounds.

The Topomer CoMFA model with good predictive ability in terms of *r*^2 ^(goodness of fit of the model) and *q*^2 ^(internal predictivity of the model) was presently developed. The model displayed a *q*^2 ^= 0.536 and *r*^2 ^= 0.975, with 0.460 standard error of prediction and 0.110 standard error of estimate. The number of components that provided the highest *q*^2 ^was eight. The summary of PLS results is provided in Table 2. The predictive ability of the developed topomer CoMFA model was assessed by the test set (eight molecules) predictions, which were excluded during the Topomer CoMFA model generation. The predictive ability of the test set was 0.604. The actual and predicted activities of the training set and test set molecules, along with the R1 and R2 fragment contributions are given in Table 3. The graph of predicted versus actual activity for training set and test set molecule is shown in Figure 2.

**Table 2:** Statistical results of Topomer CoMFA including various parameters.

N	***q***^**2**^	StdErr	***r***^**2**^	SEE	F	***r***^**2**^_**pred**_
8	0.536	0.460	0.975	0.110	105.9	0.604

N = Number of components, *q*^2^-LOO cross-validated correlation coefficient, *r*^2^-Non-cross-validated correlation coefficient, StdErr-Standard error of prediction, SEE-Standard error of estimate, F-Fischer test value, *r*^2^_pred_-Predicted correlation coefficient.

**Table 3:** Actual and predicted activities of the training set and the test set molecules with R1 and R2 fragment contributions.

			Fragment Contributions	
			
Compound	**Actual pIC**_**50**_	**Predicted pIC**_**50**_	R1	R2
4	5.39	5.23	0.39	0.40
5b	4.85	4.84	0.38	0.02
6a	5.07	4.86	0.02	0.40
7a	5.85	5.49	0.65	0.40
7b	5.25	5.11	0.65	0.02
8a	5.36	5.17	0.34	0.40
8b	4.92	4.79	0.34	0.02
9	6.24	6.27	1.44	0.40
9b	6.37	5.89	1.44	0.02
11	5.33	5.24	0.40	0.40
12	6.19	5.98	1.15	0.40
13	6.33	6.16	1.32	0.40
14	5.85	5.65	0.81	0.40
16	5.32	5.10	0.26	0.40
17	5.48	5.19	0.36	0.40
18	5.74	5.60	0.76	0.40
19	5.62	5.40	0.56	0.40
20	5.38	5.19	0.36	0.40
21	4.89	4.72	-0.12	0.40
22	5.68	5.40	0.56	0.40
25	6.17	5.97	1.14	0.40
26	5.82	5.67	0.83	0.40
27	5.51	5.50	0.66	0.40
28(temp)	6.66	6.64	1.80	0.40
30a	6.48	6.44	1.60	0.40
30b	6.11	6.06	1.60	0.02
31	6.17	6.24	1.41	0.40
32	5.82	5.78	0.94	0.40
33a	4.68	4.73	-0.11	0.40
34	4.85	4.74	-0.10	0.40
35	4.52	4.44	-0.40	0.40
**Test set**				
5a	5.27	5.22	0.38	0.40
6b	5.00	4.48	0.02	0.02
10	6.07	6.24	1.40	0.40
15	6.20	6.16	1.32	0.40
23	5.85	5.74	0.90	0.40
24	5.96	6.44	1.60	0.40
29	5.47	5.81	0.97	0.40
33b	4.72	4.35	-0.11	0.02

### Contour Map Analysis

**Figure 3:**
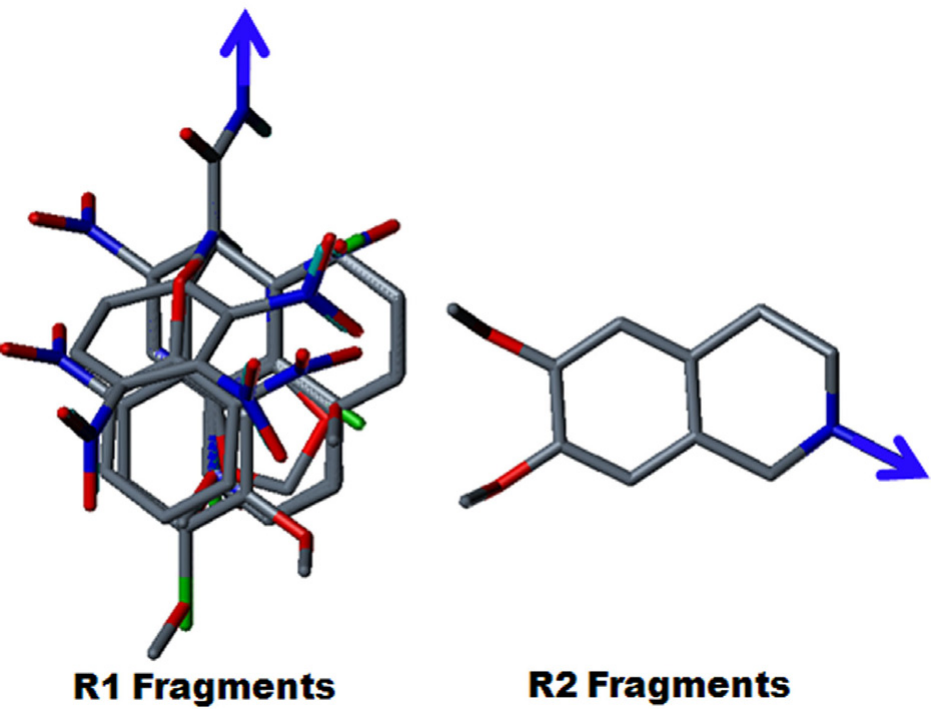
**Topological alignment of R1 and R2 fragments generated by Topomer CoMFA analysis**.

**Figure 4:**
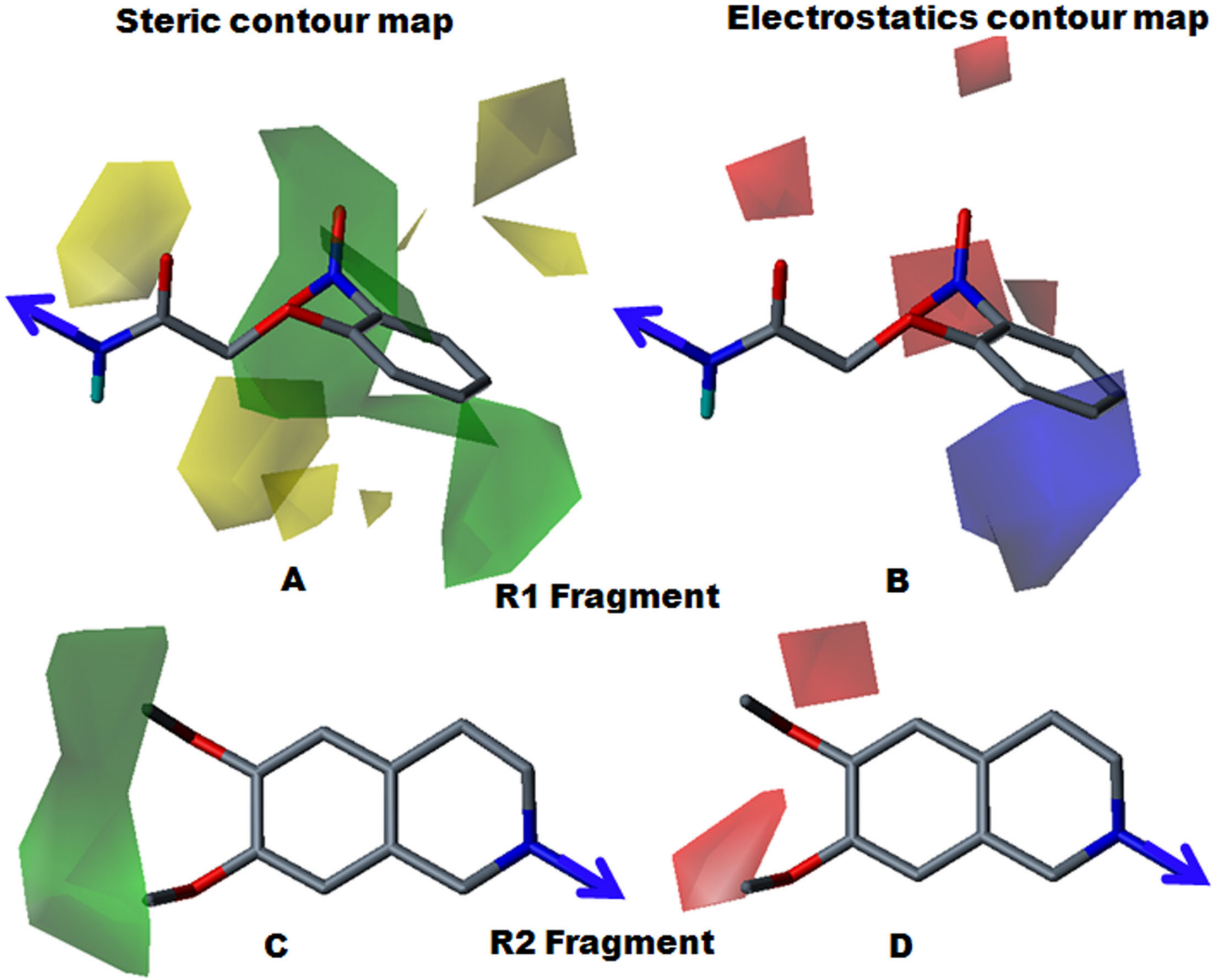
Steric and electrostatics stdev* coefficient contour map for compound 28 by Topomer CoMFA analysis. (A) Steric contour map for the R1 fragment. (B) Electrostatic contour map for the R1 fragment. (C) Steric contour map for the R2 fragment. (D) Electrostatics contour map for the R2 fragment. Sterically favoured/unfavoured areas are shown in green/yellow contour, while the blue/red polyhedra depict the favourable sites for the positively/negatively charged groups.

Topologically aligned R1 and R2 fragments are shown in Figure 3. Topomer CoMFA steric and electrostatic contour maps for the R1 and R2 fragments of the most active molecule (28) are shown in Figure 4. Contour level along with color scheme and estimated volume of contour are summarized in Table 4. In the steric contour map, the green color denotes sterically bulky groups favoured for activity and the yellow color indicates sterically bulky groups unfavoured for activity. In the electrostatics contour map, red indicates electronegative-favoured groups and blue indicates electropositive-favoured group. The steric contour map for the R1 fragment (Figure 4A) indicated that the bulky 2-nitro group on the phenoxy ring was favourable for activity. The electrostatics contour map (Figure 4B) indicated that the 2-position was favourable for polar electronegative substituent and, hence, compound 28 displayed the highest activity (pIC_50 _= 6.66) among all the compounds. In compound 29 (pIC_50 _= 5.47), the 2-nitro group of compound 28 was replaced by the 2-amino group, which produced less of an inhibitory effect. The electropositive nature of the amino group was not favourable for activity when located at the 2-position on phenyl ring, and showed decreased inhibitory potency. For compound 27 (pIC_50 _= 5.51), the unsubstituted phenyl ring was not favoured for activity; the compound displayed less inhibitory potency than compound 28. For the R2 fragment, the dimethoxy substitution was important for the inhibitory effect (Figures 4C and 4D). The green contour near the methyl group indicated that the bulky group was favoured for the inhibitory effect and the red color near the oxygen atom indicated that an electronegative substitution could retain molecular activity. The data supports the idea that the methyl group allows a hydrophobic interaction with a receptor, with an oxygen atom acting as a hydrogen-bond acceptor. This indicates the hydrophilic interaction with receptor, which echoes earlier observations [14].

**Table 4:** Topomer CoMFA contour map for the R1 and R2 fragments.

Contour		R1			R2	
	**Contour level**	**Color**	**Volume estimate**	**Contour level**	**Color**	**Volume estimate**

**Steric**	-0.014	Yellow	14.9	0.000	Yellow	0.00
	0.037	Green	29.6	0.008	Green	9.70
**Electrostatics**	-0.014	Blue	19.5	0.000	Blue	0.00
	0.032	Red	9.60	-0.004	Red	19.8

**Figure 5:**
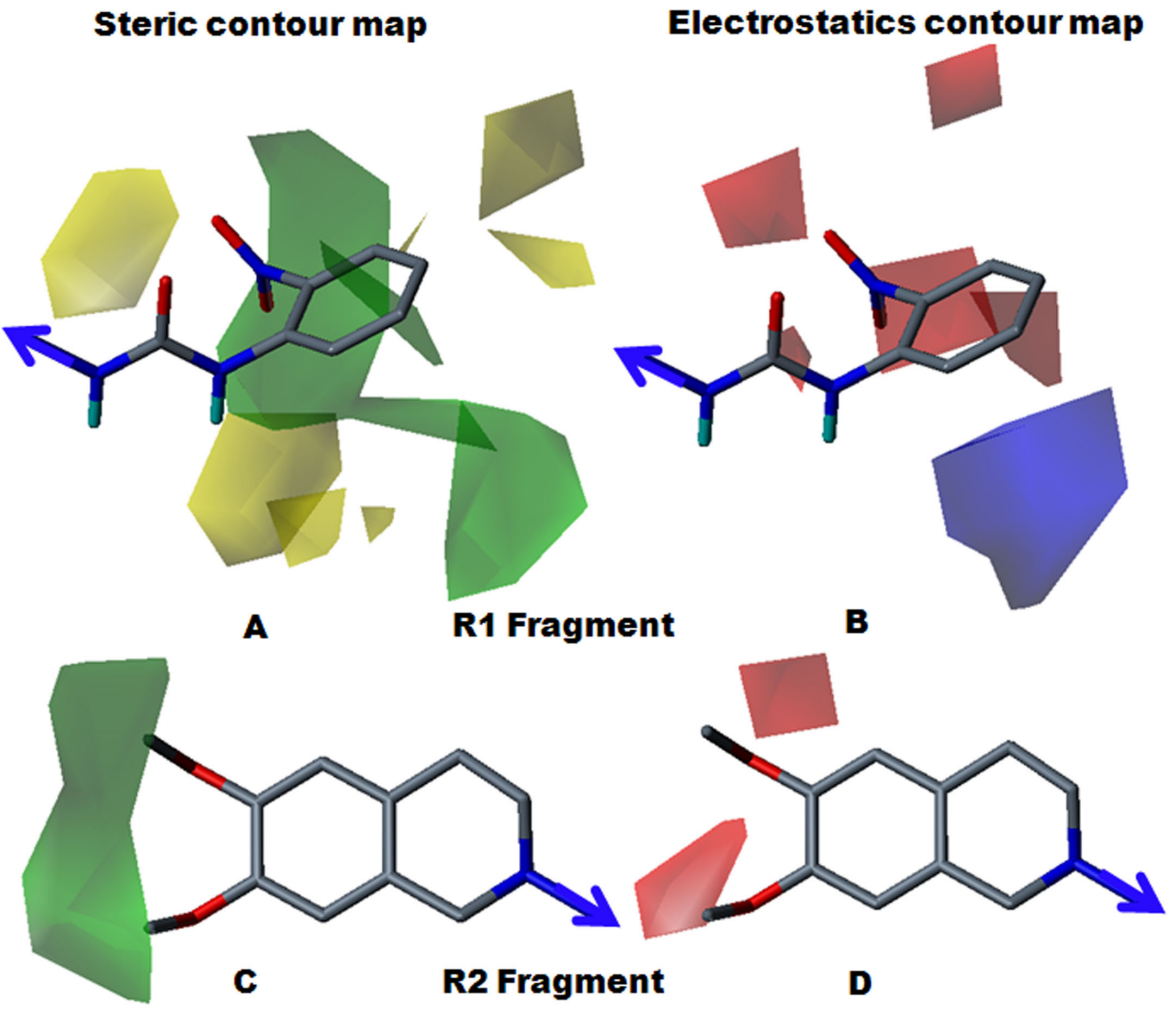
Steric and electrostatics stdev* coefficient contour map for compound 30a by Topomer CoMFA analysis. (A) Steric contour map for the R1 fragment. (B) Electrostatic contour map for the R1 fragment. (C) Steric contour map for the R2 fragment. (D) Electrostatics contour map for the R2 fragment. Sterically favoured/unfavoured areas are shown in green/yellow contour, while the blue/red polyhedra depict the favourable site for the positively/negatively charged groups.

Steric and electrostatic contour maps for compound 30a (pIC_50 _= 6.48) are shown in Figure 5. Fragment R1 consisted of a urea substituted derivative. The steric contour (Figure 5A) revealed that a phenyl ring with a 2-nitro substitution is favoured for activity. The electrostatic contour map (Figure 5B) indicated that the electron withdrawing nature of the nitro group was favourable for increasing inhibitory potency. For compound 30b (pIC_50 _= 6.11), removal of the dimethoxy group from the R2 fragment produced a decreased activity compared to compound 30a, indicating the necessity of the dimethoxy substitution for the inhibitory effect. For compound 31 (pIC_50 _= 6.11), and 32 (pIC_50 _= 5.82), the position of nitro group played a major role in the inhibitory effect, as a para-substituted phenyl ring (compound 32) displayed less inhibitory potency than a meta-substituted phenyl ring (compound 31). The yellow contour corresponding to the para position of the phenyl ring indicated that a bulky group at this position was unfavourable for activity, perhaps due to the steric restriction of the receptor pocket. For compound 33a (pIC_50 _= 4.68), 34 (pIC_50 _= 4.85) and 35 (pIC_50 _= 4.51), amino group substitutions at the ortho, meta and para positions led to the least-active compounds, indicating that, for P-gp inhibitory effect, a polar electronegative group (nitro) was more favourable than an electropositive group (amino).

**Figure 6:**
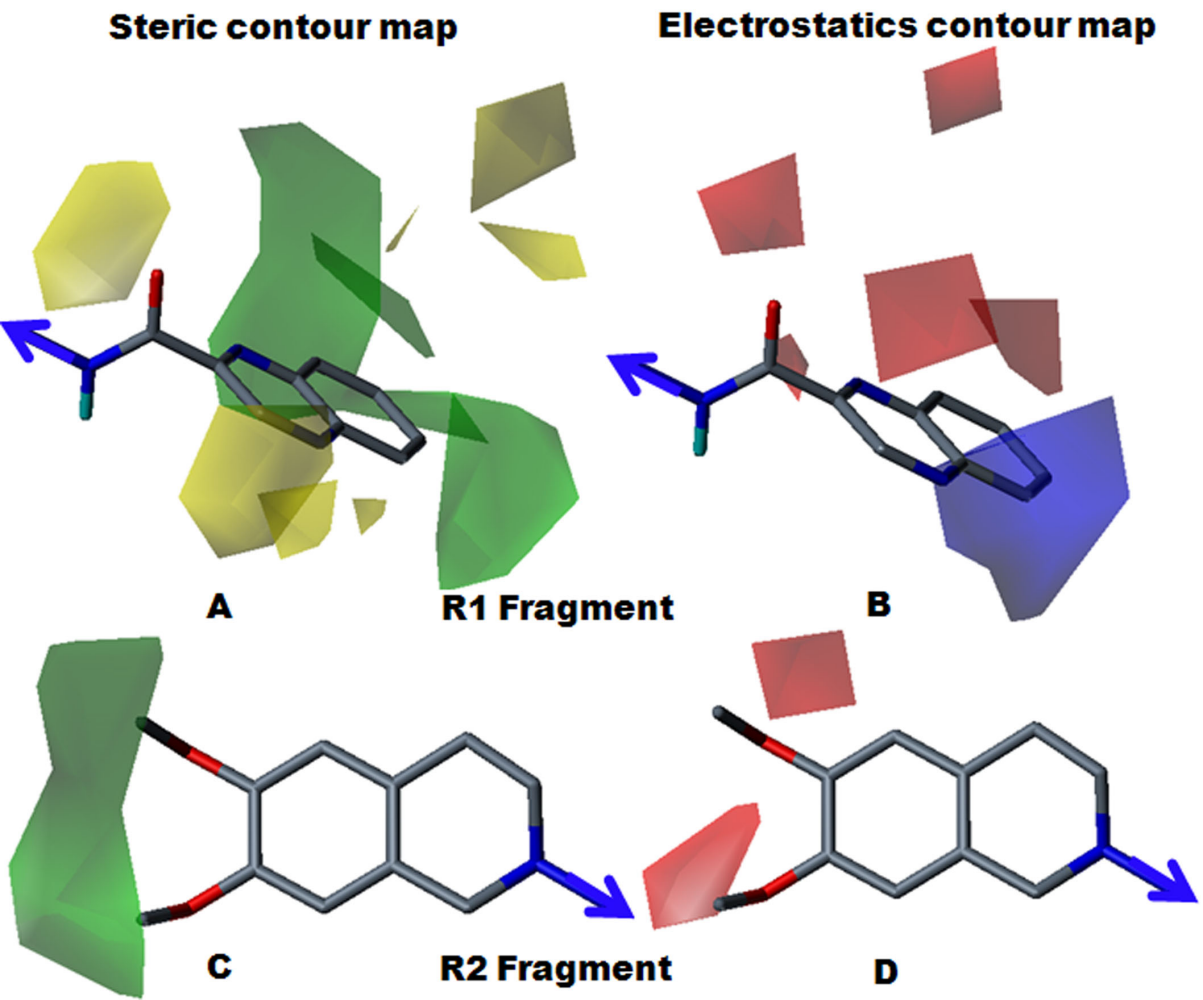
Steric and electrostatics stdev* coefficient contour map for compound 13 by Topomer CoMFA analysis. (A) Steric contour map for the R1 fragment. (B) Electrostatic contour map for the R1 fragment. (C) Steric contour map for the R2 fragment. (D) Electrostatics contour map for the R2 fragments. Sterically favoured/unfavoured areas are shown in green/yellow contour, while the blue/red polyhedra depict the favourable site for the positively/negatively charged groups.

Steric and electrostatic contour maps for compound 13 (pIC_50 _= 6.33) are shown in Figure 6. Fragment R1 consisted of an amide-substituted derivative. The steric contour map (Figure 6A) revealed that the bulky nature of the quinoxalinyl ring was favourable for inhibitory potency. The electrostatics contour map (Figure 6B) revealed that the electropositive nature of quinoxalinyl ring was favoured for increased inhibitory potency. Replacement of the quinoxalinyl ring by a 2-naphthyl ring in compound 15 (pIC_50 _= 6.20) slightly decreased the inhibitory potency. Compound 13 was more electropositive than compound 15, because the two lone pairs of nitrogen atoms in the quinoxalinyl ring increased the electropositivity of the ring, boosting the inhibitory potency of compound 13, relative to compound 15. Removal of a dimethoxy group from the R2 fragment decreased the inhibitory potency. For compound 8b (pIC_50 _= 4.92), removal of the dimethoxy groups produced the least potent compound.

**Figure 7:**
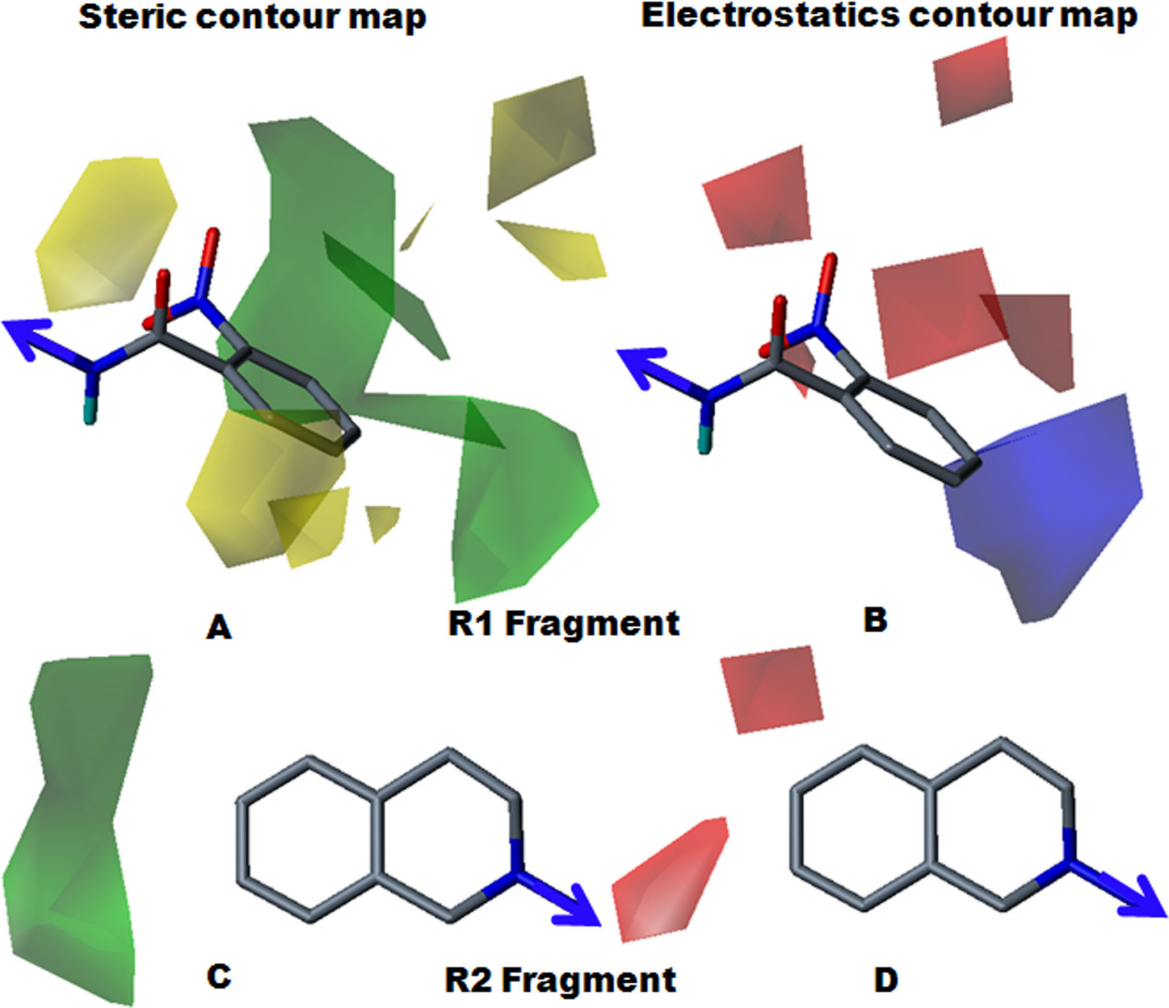
Steric and electrostatics stdev* coefficient contour map for compound 5b by Topomer CoMFA analysis. (A) Steric contour map for the R1 fragment. (B) Electrostatic contour map for the R1 fragment. (C) Steric contour map for the R2 fragment. (D) Electrostatics contour map for the R2 fragment. Sterically favoured/unfavoured areas are shown in green/yellow contour, while the blue/red polyhedra depict the favourable site for the positively/negatively charged groups.

Steric and electrostatic contour maps for compound 5b (pIC_50 _= 4.85) are presented in Figure 7. The steric contour map for fragment R1 (Figure 7A), which displayed a yellow contour near the 2-nitro position, was indicative of the lower favourability of that particular position for the inhibitory effect. The electrostatic contour map for the R1 fragment (Figure 7B), which displayed a red contour near the nitro groups, indicated that the position was favorable for an electronegative group. For compound 17 (pIC_50 _= 5.48), compound 18 (pIC_50 _= 5.78) and compound 19 (pIC_50 _= 5.62), substitutions with the less bulky and electronegative bromine at ortho, meta and para positions improved the potency over compound 5b. Fragment R2 (Figures 7C and 7D) displayed green and red contours near positions 6 and 7 of the 3,4-dihydroisoquinoline ring, indicating the importance of the dimethoxy group for the inhibitory effect. In contrast, the presence of a dimethoxy group in compound 5a (pIC_50 _= 5.27) improved potency over compound 5b.

### HQSAR analysis

**Figure 8:**
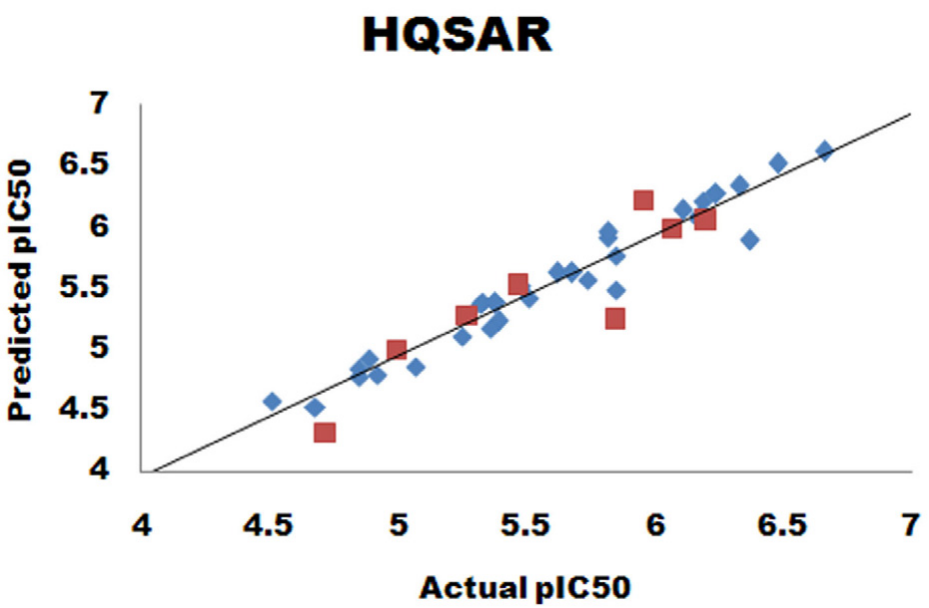
**Scatter plot diagram for HQSAR analysis**. The scatter plot displays the predicted versus actual activities of the training set (blue diamond) and the test set (red square) of compounds.

The HQSAR model with good predictive ability in terms of *r*^2 ^and *q*^2 ^was presently developed. The model shows *q*^2 ^= 0.777 and *r*^2 ^= 0.956 with 0.302 standard error of prediction and 0.105 standard error of estimate. The model was developed with bond (B), connection (C) and donor acceptor (DA) parameters with BHL = 97. The number of components that provided the highest *q*^2 ^was six. Table 5 summarizes the PLS results. The predictive ability of the developed HQSAR model was assessed by the test set (eight molecules) predictions, which were excluded during HQSAR model generation. The predictive ability of the test set was 0.730. The actual and predicted activities of the training set and test set molecules are given in Table 6. The graph of predicted versus actual activities for the training set and test set molecules is shown in Figure 8.

**Table 5:** Statistical results of HQSAR including various parameters.

	N	**q**^**2**^	StdErr	**r**^**2**^	SEE	***r***^***2***^_**pred**_	BHL
**HQSAR**	6	0.777	0.302	0.973	0.105	0.730	97

N = Number of components, q^2^-LOO cross-validated correlation coefficient, r^2^-Non-cross-validated correlation coefficient, StdErr-Standard error of prediction, SEE-Standard error of estimate, r^2^_pred_-Predicted correlation coefficient, BHL-best hologram length.

**Table 6:** Actual and predicted activities for the training set and the test set by HQSAR model.

Compound	**Actual pIC**_**50**_	**Predicted pIC**_**50**_
4	5.39	5.23
5b	4.85	4.84
6a	5.07	4.86
7a	5.85	5.49
7b	5.25	5.11
8a	5.36	5.17
8b	4.92	4.79
9	6.24	6.27
9b	6.37	5.89
11	5.33	5.39
12	6.19	6.21
13	6.33	6.34
14	5.85	5.77
16	5.32	5.37
17	5.48	5.51
18	5.74	5.57
19	5.62	5.63
20	5.38	5.38
21	4.89	4.92
22	5.68	5.63
25	6.17	6.10
26	5.82	5.92
27	5.51	5.42
28(temp)	6.66	6.62
30a	6.48	6.52
30b	6.11	6.14
31	6.17	6.07
32	5.82	5.96
33a	4.68	4.53
34	4.85	4.78
35	4.52	4.58
**Test set**		
5a	5.27	5.27
6b	5.00	4.99
10	6.07	5.98
15	6.20	6.06
23	5.85	5.25
24	5.96	6.21
29	5.47	5.53
33b	4.72	4.31

Numerous models with combinations of the A/B/C/H/Ch/DA parameters with 4-7 atom counts are given in Table 7. Parameters such as B/C/DA were important for model generation. Bond and connection considered the bond order and hybridization states within fragments, respectively, and DA yielded information about the donor and acceptor atoms. The highest *q*^2 ^value was obtained for parameters B/C/DA; for further improvement of *q*^2^, an optional atom count (1-10) was explored. A significant difference was noticed in the statistical parameters with different atom counts for model B/C/DA (Table 8).

**Table 7:** Different statistical parameter obtained for HQSAR models.

Model	Fragment distinction	N	***q***^**2**^	StdErr	***r***^**2**^	SEE	BHL
1	A/B	6	0.432	0.482	0.908	0.194	353
2	A/DA	6	0.504	0.45	0.935	0.162	199
3	B/DA	6	0.671	0.367	0.951	0.142	97
4	A/B/C	6	0.658	0.374	0.945	0.149	151
5	A/B/Ch	6	0.661	0.372	0.945	0.150	151
6	A/B/DA	6	0.731	0.332	0.961	0.125	353
**7**	**B/C/DA**	**6**	**0.772**	**0.305**	**0.956**	**0.134**	**97**
8	A/B/C/Ch	6	0.661	0.372	0.945	0.150	151
9	A/B/H/DA	6	0.713	0.342	0.958	0.130	257
10	A/B/Ch/DA	6	0.733	0.33	0.963	0.122	353
10	A/B/C/H/Ch	6	0.375	0.505	0.910	0.192	307
11	A/B/C/Ch/DA	6	0.705	0.347	0.966	0.118	151
12	A/B/C/H/DA	6	0.676	0.364	0.959	0.130	307
13	A/B/H/DA/Ch	6	0.704	0.348	0.957	0.132	257
14	A/B/C/H/DA/Ch	5	0.603	0.395	0.894	0.204	151

A-atom; B-bond; C-connection; Ch-chirality; DA-donor/acceptor; Best model is highlighted in **bold font**.

**Table 8:** Statistical parameters obtained for model 7 with different atom counts.

Atom Count	N	***q***^**2**^	StdErr	***r***^**2**^	SEE	BHL
1-4	6	0.511	0.447	0.871	0.230	151
2-5	6	0.636	0.385	0.900	0.202	307
3-6	5	0.683	0.352	0.950	0.141	353
4-7	6	0.772	0.305	0.956	0.134	97
**5-8**	**6**	**0.777**	**0.302**	**0.973**	**0.105**	**97**
6-9	4	0.726	0.321	0.948	0.140	353
7-10	6	0.770	0.306	0.979	0.092	307

The final model chosen for HQSAR analysis is highlighted in **bold font**.

A standard color coding system was used to indicate atomic contributions in the HQSAR model. Red, red-orange and orange designated unfavourable and negative contribution to the activity, while yellow, green-blue and green denoted favourable or positive contribution to the activity. White indicated an intermediate contribution to activity. For study of atomic contribution, molecules were selected randomly. The positive and negative atomic contributions for the selected molecules are shown in Additional file 1. All the molecules in the dataset had a common substructure and varied only in R_1 _and R_2 _substructure. The contribution map for compound 12 showed that the R_2 _side chain contributed positively to activity (pIC_50 _= 6.17), whereas the remainder displayed an intermediate contribution to activity. The contribution map for compound 13 (pIC_50 _= 6.33) indicated that the quinoxalin-2-carboxamide substituent contributed positively to the inhibitory effect. This result was consistent with the Topomer CoMFA results. The contribution map for compound 19 (pIC_50 _= 5.62) indicated that the R_2 _side chain contributed in an intermediate fashion to activity. But, the central phenyl ring contributed positively to activity. For compound 26 (pIC_50 _= 5.82), the contribution map revealed that [(4, 5-dimethoxy-2-nitrophenyl)-acrylamide] substituents contributed positively to activity, while the remainder of the structure contributed in an intermediate fashion to the inhibitory effect. The highly active compound 28 of the series (pIC_50 _= 6.66) contributed in a positive and intermediate manner to activity. The R_2 _side chain (2-nitrophenoxy-acetamide) substitution contributed in an intermediate manner. The central phenyl ring and isoquinoline ring displayed a positive contribution to activity. Similarly, another highly active compound in the series, 30a (pIC_50 _= 6.48), displayed similar results. The R_2 _side chain consisting of 2-nitrophenylurea substituents contributed moderately to activity, and the central phenyl and isoquinoline rings contributed fairly positive to activity. Compound 7b (pIC_50 _= 5.25) showed a moderate contribution to activity and compound 9b (pIC_50 _= 6.37) contributed moderately and positively to the inhibitory effect.

**Figure 9:**
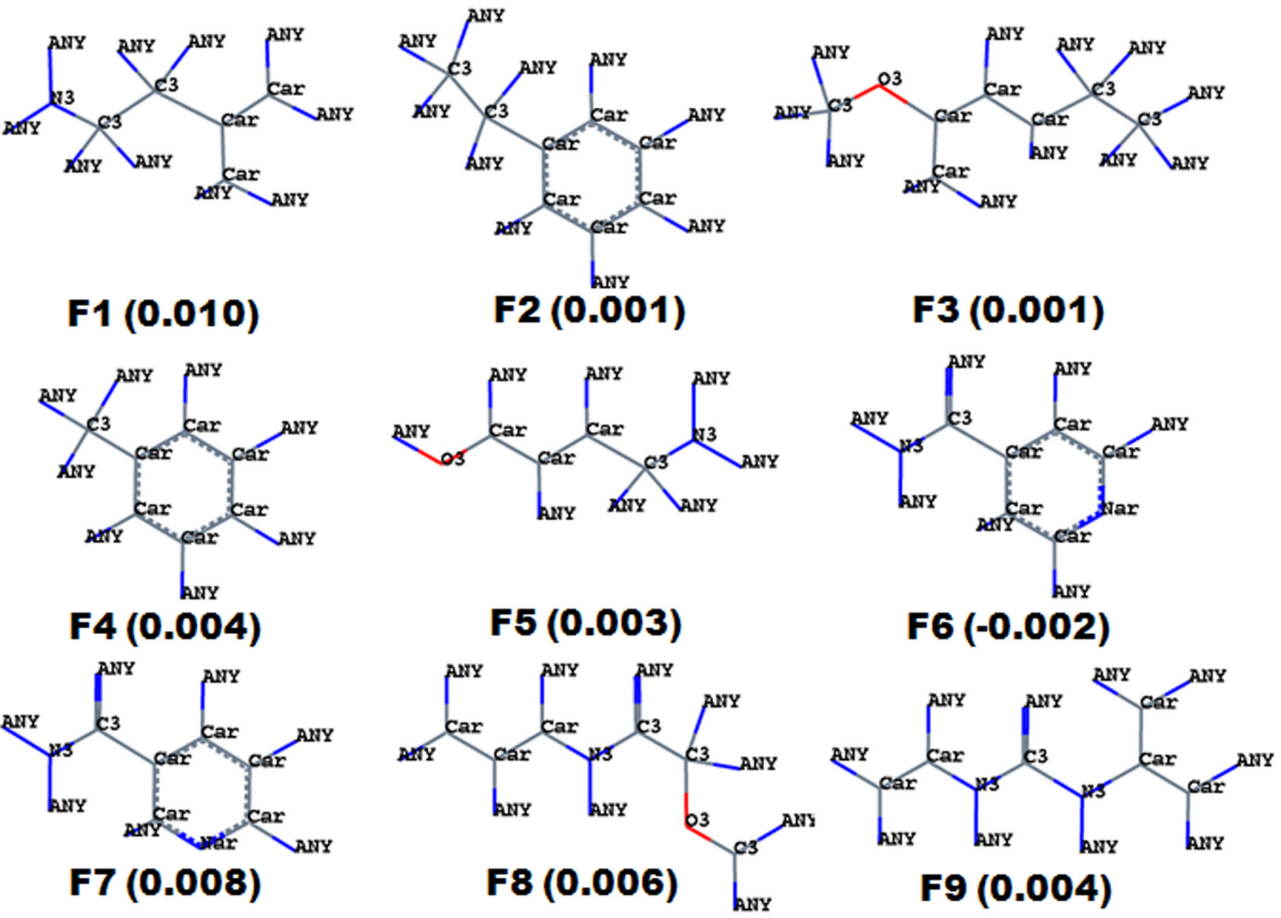
**Positive and negative contribution of some fragments towards P-gp antagonism, obtained by HQSAR analysis**. Grey = carbon atoms, where C2, C3 and Car indicates sp^2^, sp^3 ^and aromatic carbon. Red = oxygen atom, where O2 and O3 indicates sp^2 ^and sp^3 ^oxygen, respectively. Blue = nitrogen atom, where N3, Nar indicates sp^3 ^and aromatic nitrogen. Blue = ANY atom, and it may be hydrogen, carbon or oxygen.

HQSAR mainly deals with the fragments; the final HQSAR model with B/C/DA parameters generated thousands of fragments. Correlation of each fragment with biological activity was impossible. Instead, the fragments produced by HQSAR were analyzed. This analysis shed light on some of the important features; some representative molecular fragments are displayed in Figure 9. The fragment analysis indicated that fragments possessing positive values contributed favourably to activity, while fragments possessing negative values contributed unfavourably to activity. Fragment F1, which had a positive coefficient value of 0.010, possessed tertiary nitrogen connected to the aromatic ring with ethyl linker. This fragment was present in all molecules of the data set, indicating the importance of tertiary nitrogen for the inhibitory effect. This fragment may be preferred when designing a new scaffold for P-gp antagonism. Fragment F2, which contained an ethyl phenyl ring with a coefficient value of 0.001, was also present in all molecules of the dataset. Fragment F3, which had the same coefficient value, was present in some molecules. Fragment F3 contained a methoxy group on the aromatic ring; the fragment plays an important role in a favourable hydrophobic interaction, consistent with previous results [14]. The oxygen residue of the methoxy group acts as a hydrogen bond acceptor, which supports hydrophilic interaction with receptor, also consistent with previous observations [14]. In this particular series of compounds, the tetrahydroisoquinoline moiety was either unsubstituted or was substituted by a 6, 7-dimethoxy group. The difference in inhibitory effect of the dimethoxy substituted and unsubstituted compounds suggest that this substructure might be important in the inhibitory effect. Fragment F4 displayed a positive contribution of 0.004 for activity and was present in all molecules as a central phenyl ring. It might act through hydrophobic interactions with receptors, as has been previously suggested [13]. Fragment F6 was present in molecule 11 (pIC_50 _= 5.33) and contributed negatively for activity, with a coefficient of -0.002. This result indicates that the 4-quinoline substituent contributes negatively to activity and decreases inhibitory potency. Fragment F7 consisted of a 3-quinoline nucleus and was present in compound 9 (pIC_50 _= 6.24) and 9b (pIC_50 _= 6.37); the fragment contributed positively to activity (coefficient of 0.008). The aromatic (3-quinolinyl) 'N' acts as a hydrogen-bond acceptor with the appropriate amino acid of the receptor [14]. Fragment F8 was present in molecules 27 (pIC_50 _= 5.51), 28 (pIC_50 _= 6.66) and 29 (pIC_50 _= 5.47), where it contributed positively to the inhibitory effect (coefficient of 0.006); these results indicate that an ether linker is more important for an inhibitory effect. Fragment F9 was present in the urea derivative compound; its' contribution coefficient of 0.004 was indicative of an inhibitory effect, highlighting the importance of urea substituted derivatives for the inhibitory effect.

The model generated by both Topomer CoMFA and HQSAR agreed well with each other. The HQSAR analysis showed that tertiary nitrogen with an ethyl phenyl linker was essential for activity and that a dimethoxy group was necessary in inhibition of P-gp. Urea and ether linker were most important for the inhibitory effect and contributed profoundly. The fragment from the isoquinoline ring was also vital for activity and for the inhibitory effect.

Klinkhammer paper is about structure activity relationship (SAR) and this manuscript describes quantitatively structure activity relationship (QSAR), which is consistent with the previous paper. The present approach is in-depth study, i.e., contour map analyses of CoMFA and fragment analyses of HQSAR provided guidelines concerning compound modification.

The presently-developed QSAR models yielded similar findings as XR9576 reports [26]. The third-generation MDR modulators have the same scaffold as XR9576, with different side chains. The sterically bulky side chain of XR9576 could be responsible for the higher inhibitory effect [26]. This view is supported by the present findings of favourable steric (green) and electrostatic (blue) contour maps around the R1 fragment. It also highlights the importance of fused benzo rings. In the current model, the fused ring containing compounds (such as quinolinyl, quinoxalinyl and naphthyl) are predicted to show higher activity. The XR9576 report also highlighted the importance of the positional effect of nitrogen in quinolinyl ring for inhibitory potency [26]. This positional effect was presently confirmed quantitatively.

### Designing New Compounds

Ligand-based methods such as Topomer CoMFA are not computationally intensive and can lead to the rapid generation of QSARs, from which the biological activity of newly designed compounds can be predicted. In contrast, an accurate prediction of activity of untested compounds based on the computation of binding free energies is both complicated and lengthy. The Topomer CoMFA contour maps provide clear indicators for designing novel molecules with improved P-gp inhibitory potency. The careful analyses of contour maps and HQSAR results led to the identification of the structural requirements responsible for compounds having improved potency. The information obtained from the contour maps of the most potent molecule (28; Figure 4) was utilized to design new R1 fragment containing compounds.

**Figure 10:**
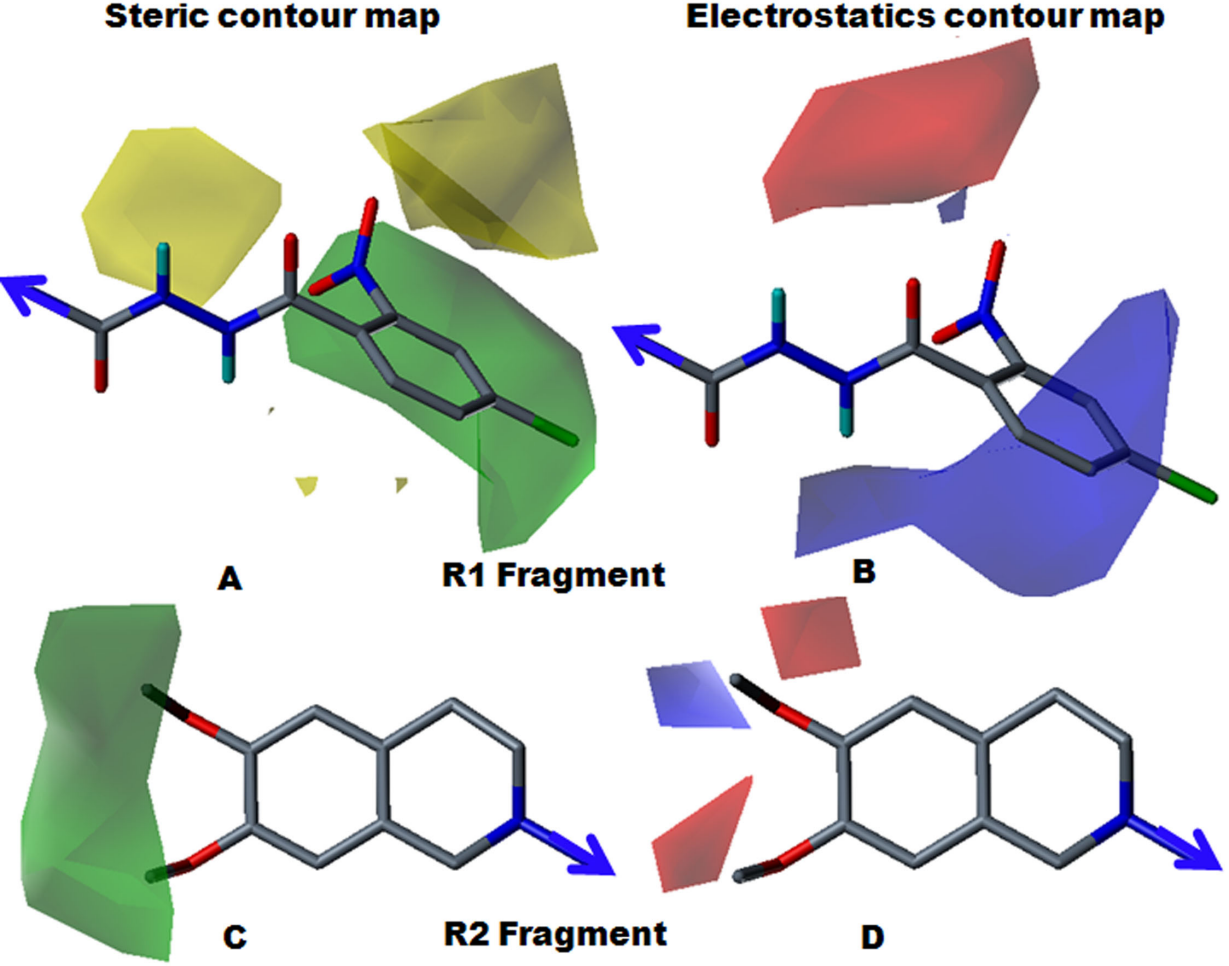
Steric and electrostatics stdev* coefficient contour map for designed compound 28B by Topomer CoMFA analysis. (A) Steric contour map for the R1 fragment. (B) Electrostatic contour map for the R1 fragment. (C) Steric contour map for the R2 fragment. (D) Electrostatics contour map for the R2 fragment. Sterically favoured/unfavoured areas are shown in green/yellow contour, while the blue/red polyhedra depict the favourable site for the positively/negatively charged groups.

It was assumed that the 2-nitro group on the phenyl ring is favourable and responsible for retaining higher potency of inhibitors. The electrostatically favourable blue color at the para position of the 2-nitrophenoxy ring indicates that substituents with an electron-rich group at this position might increase the activity. Presently, a chlorine group was substituted at the para position and the phenoxy-acetamide group was replaced by *N*'-keto-benzohydrazide group. The resulting compound (28B) displayed improved inhibitory potency over compound 28. Here, using information obtained from the Topomer CoMFA model, five new R1 fragment containing compounds were designed, which displayed significant increases in activity. These newly designed compounds were included in the test set and their activity was predicted by the Topomer CoMFA and HQSAR models (Table 9). The Topomer CoMFA contour map was predicted for the designed compound 28B shown in Figure 10. The (*E*)-*N*-3-(2-nitrophenyl)acrylamide (R1 fragment of compound 24) was replaced by a (*2Z,4E*)-5-(2-nitrophenyl)penta-2,4-dienenitrile (28A fragment) substituent, which resulted in equipotent compound as 28. The 2-nitrophenyl group remained unchanged because of its delicacy and importance at particular position for inhibitory effect, which corresponded to the sterically and electrostatically favourable contour maps.

**Table 9:** Designed molecular fragments along with their predicted activities by Topomer CoMFA and HQSAR analysis.

Molecule number	R1 Fragments	Predicted Activity	
		
		Topomer CoMFA	HQSAR
28A		6.66	6.62
28B		6.94	6.78
28C		6.74	6.79
28D		6.60	6.52
28E		6.57	6.34

For all designed molecules, the variable part is the R1 fragment.

Another highly potent compound 28 derivative (28C) was designed by replacing the 2-nitrophenoxy-acetamide group of compound 28 by 2-(5-(4-chlorocyclohexyl)-1-methyl-1*H*-imidazol-2ylthio)-*N*-acetamide. Here, the essential 2-nitrophenyl group was replaced by methyl substituted imidazole group by considering its positional and steric effects. The sterically favourable bulky nature of the nitro group was replaced by a methyl group, which corresponded to the favourable green contour map. The fifth position of the imidazole ring was substituted with a 4-chlorocyclohexyl group. The electron-rich chloro group at this position was favourable for improved inhibitory potency, which corresponded to the blue contour map in the vicinity.

To check the importance of ortho substituents on the inhibitory effect, the nitro group was removed from the ortho position of compound 28. Decreased inhibitory potency was observed in compound 28D, indicating that retention of inhibitory potency of an inhibitor requires substitution of the phenyl ring with an ortho nitro group. Steric and electrostatic contour maps for the R2 fragment of 28B are displayed in Figure 10, and demonstrate the importance of a sterically bulky methoxy group for the inhibitory effect. We analyzed individual atomic contribution map of newly designed molecules for inhibitory effect. It indicates that the R1 fragment of designed molecules contribute positively towards the inhibitory effect, as denoted by the blue, green-blue and yellow color in HQSAR.

In summary, utilizing information obtained from the Topomer CoMFA and HQSAR analyses, we designed novel fragment containing compounds, which had higher inhibitory potency than the reported compound. From the overall analyses, we conclude that the 2-nitrophenyl group, which has a steric as well as polar nature, is responsible for the higher affinity of the molecules. Additionally, designed fragments underscore the importance of electron-rich substituents at the para position of the phenyl and cyclohexyl ring system.

## Conclusion

We derived Topomer CoMFA and HQSAR models with good statistical values. The robustness of these models was confirmed using a test set. Topomer CoMFA analysis provided great insight into the structural requirements for improved potency over existing compounds. The information obtained from HQSAR model shows the importance of bond, connection and donor/acceptor parameters. The overall study indicates that, in HQSAR analysis, fragments containing information about the dimethoxy group are important for an inhibitory effect; this was supported by the findings of the Topomer CoMFA contour map. The contour map for ether and urea linking fragments (R1) indicate that substitution of bulkier and polar group to the ortho position of benzene ring enhances the inhibitory effect, and explains why the compounds with nitro group have good inhibitory potency. Contour map analysis also revealed that bulky and more electropositive substituents on the amide linker are responsible for higher potency; this was supported by the HQSAR atomic contribution map. A central phenyl ring could hydrophobically interact with a receptor and so is important in the inhibitory effect. In summary, both HQSAR and Topomer CoMFA underscore the importance of the aromatic dimethoxy and nitro groups for the inhibitory effect. Molecular modeling techniques like Topomer CoMFA and HQSAR aid the identification of the functional groups and atoms important for the inhibitory potency. Together, these data can be utilized to design more potent compounds than the present series of compounds.

## Authors' contributions

CGG designed the experiments, carried out all computational work, analyzed results and wrote the manuscript. SJC, TM and GK analyzed the results and discussed scientific points. SJC also designed the experiments. All authors read and approved the final manuscript.

## Supplementary Material

Additional file 1 **Contribution map**. Positive and negative contribution map for few molecules obtained by HQSAR analysis. Click here for file
