# Supplementary material for: In Silico Quantitative Structure-Activity Relationship Studies on P-gp Modulators of Tetrahydroisoquinoline-Ethyl-Phenylamine Series
Source: BMC Struct Biol. 2011 Jan 26;11:5. doi: 10.1186/1472-6807-11-5 (PMC3038138; doi:10.1186/1472-6807-11-5)

Additional file 1. Contribution map: Positive and negative contribution map for few molecules obtained by HQSAR analysis.

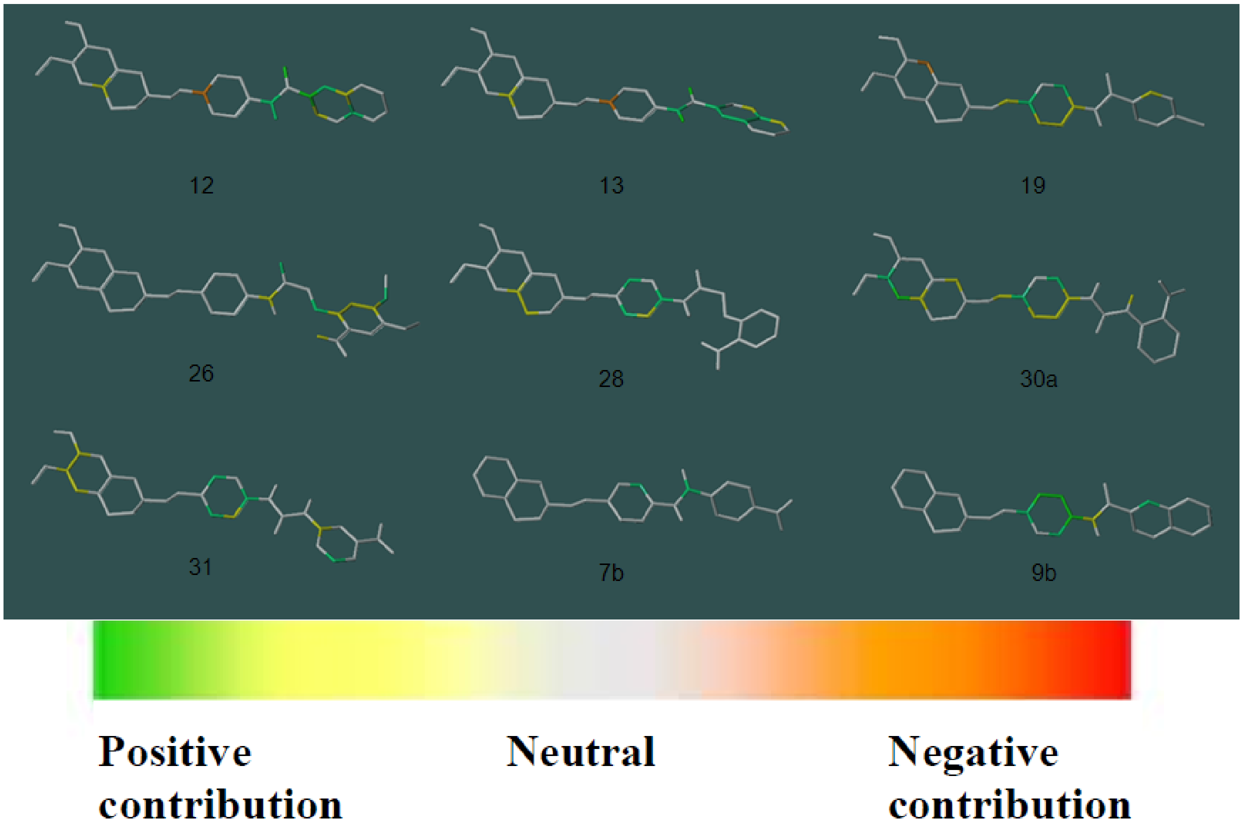

Supplement: Additional file 1 — Contribution map. Positive and negative contribution map for few molecules obtained by HQSAR analysis. [file 1472-6807-11-5-S1.PDF]
